# Impacts of COVID-19 on tourism and management response from Banff National Park, Canada

**DOI:** 10.1007/s11676-022-01580-4

**Published:** 2023-02-01

**Authors:** Christina Dehui Geng, Howie W. Harshaw, Wanli Wu, Guangyu Wang

**Affiliations:** 1 Department of Forest Resources Management, Faculty of Forestry, University of British Columbia, Vancouver, Canada; 2 Faculty of Kinesiology, Sport, and Recreation, University of Alberta, Edmonton, Canada

**Keywords:** National park, COVID-19 pandemic, Visitor experience, Tourism management, Visitor perception

## Abstract

The COVID-19 pandemic posed challenges to the tourism sector globally. We investigated changes in visitor demographics, satisfaction level, and its determinants pre- and peri-COVID-19. Data were collected using questionnaire surveys in 2019 and 2021 within Banff National Park (BNP). The data analyses were based on a sample size of 1183 respondents by conducting factor analysis, correlation analysis and stepwise regression analysis. Results highlight that there were fewer international visitors and more local and domestic visitors during the pandemic. Park attributes were evaluated at a higher satisfaction level peri-COVID-19. The quality of the Park facilities and services were the most important satisfaction determinants pre- and peri-COVID-19, and all the Park COVID-19 measures and actions received positive experience from visitors. This research fills this knowledge gap by developing a better understanding in the change of visitor demographics and satisfaction level in BNP under the context of the pandemic. It also provides implication for both scholars and practitioners to understand the impacts of the pandemic on Park visitation. The study can provide insights for utilizing the pandemic as a transformative strength and for mitigating its negative impact on tourism industry.

## Introduction

### Impacts of COVID-19 on tourism in national parks.

The COVID-19 global pandemic has created unprecedented public health, economic, social, and environmental challenges (WHO 2020; Waithaka et al. 2021). Most national parks worldwide closed their visitor facilities and services, suspended park operations and activities, and limited social gatherings and events at the beginning of the pandemic (Hockings et al. 2020; Town of Banff 2020; Waithaka et al. 2021). Lockdown regulations and travel restrictions significantly affected park visitation, tourism revenues, local stakeholder and community engagement, and park management capacity (Hockings et al. 2020; Kupfer et al. 2021; Mandić 2021; Smith et al. 2021; Spenceley et al. 2021).

The impacts of COVID-19 on national park visitation and associated tourism sectors varied globally, and present opportunities to investigate national park management approaches (Kupfer et al. 2021; Waithaka et al. 2021). COVID-19 associated responses such as restrictions on social gatherings and internal movement, stay at home policy and event cancellations increased people’s demand for natural environment and outdoor recreation opportunities (Geng et al. 2021). These changes have highlighted the important and irreplaceable roles that nature plays on human physical and psychological health.

### Impacts of COVID-19 on Banff National Park visitation.

As Canada’s oldest national park, Banff National Park, Alberta, is a tourist destination that has been affected by the COVID-19 pandemic. The number of Park visitors has increased since 2010; by 2019, the number had reached 4.2 million, making it the most visited national park in Canada and the third most visited park in North America (Parks Canada 2019; Statista 2020). Parks Canada announced on March 19, 2020, that visitor facilities, services and vehicle access in BNP were temporarily closed to reduce the potential risks to visitors and local employees and limit the spread of COVID-19 (Town of Banff 2020); as a result, there were no Park visitors in April and May 2020. The Park’s closure caused the loss of tourism-related jobs. The collapse of international tourism and related income negatively impacted local gateway communities (Town of Banff 2020; Ellis 2021; Waithaka et al. 2021).

On June 1, 2020, limited visitor access and day-use areas with capacity control resumed in BNP, resulting in a significantly increased number of visitors. The number of visitors in July and in August 2020 was only 15% lower than the previous year. However, before COVID-19 started, Banff National Park attracted four million visitors annually and more than 50% of the visitors are from outside of Canada (CBC 2020). Under the international and internal travel restrictions in 2020, indicating that there were more domestic visitors especially local visitors chose to visit Banff National Park. People’s response to government COVID-19 pandemic policies revealed the irreplaceable role of parks to public health and well-being (Hockings et al. 2020; Geng et al. 2021): more people were accessing nearby parks and green spaces to seek out nature connections to address the mental and physical health consequences of isolation policies, especially under the closure of shopping malls, restaurants and other public places at the beginning of the pandemic. This highlighted the importance of investigating the management strategies that seek to improve visitors’ experiences, and an examination of visitors’ perceptions of COVID-19 measures employed in BNP to mitigate the economic, social, and environmental impacts of COVID-19.

### Importance of managing visitor experience and satisfaction under the COVID-19 pandemic.

Visitor satisfaction has been framed in terms of the behavioral model of outdoor recreation, which is based on expectancy theory (i.e., human behavior is goal-oriented; Manning 2011). Satisfaction is one of the most important sources of competitiveness in tourism destinations (Yuan et al. 2008), and an indicator of the quality of park services, facilities, and natural landscape (Yuksel 2001; Tian-Cole et al. 2002; Rodger et al. 2015; Hilsendager et al. 2017; Thapa and Lee 2017; Agyeman et al. 2019; Fossgard and Fredman 2019). Visitor experience is an important element of national park management: high levels of visitor satisfaction can lead to visitor loyalty, community support, economic development, and tourism sustainability (Gursoy et al. 2007; Neal and Gursoy 2008; Frost and Hall 2009; Rodger et al. 2012; Amin et al. 2014). Therefore, external and internal forces that determine and shape visitors’ overall satisfaction, and tourism development in national parks need to be investigated to achieve or maintain the competitiveness and sustainability of the tourism sector (Ryan et al. 2010; Chen et al. 2011).

Measuring visitor satisfaction provides information about whether their expectations were met, and guides the management and planning of visitor services, facilities, and infrastructure. In the context of the COVID-19 pandemic, tourism experience in national parks became more important for several reasons. First, the COVID-19 pandemic highlighted the dependency of national park-local/gateway communities on tourism (Spenceley 2021). For example, 90% of the Town of Banff’s economy was generated from tourism, especially from international visitors (Mertz 2020). At the beginning of the pandemic, 80% − 85% of town employees were laid off due to the subsequent lockdown and no visitors. Therefore, managing and ensuring visitor safety and experiences in national parks will continue to support local economies and contribute to sustainable tourism.

Secondly, health restrictions due to COVID-19 changed people’s lifestyles. Health crisis exaggeration from social media, and COVID-19 related financial and food insecurities posed negative physiological and psychological impacts on people (Wu et al. 2005; Monson et al. 2017; Bo et al. 2021; Brooks et al. 2020; Gao et al. 2020; Xiang et al. 2020; Geng et al. 2021). During this period, national parks received renewed attention by providing safe, open spaces for people to pursue outdoor activities, mitigate the adverse effects of COVID-19 by providing access to natural elements such as serenity, space, wildness, and environment (Seaman et al. 2010; Annerstedt et al. 2012; Hockings et al. 2020; Nicola et al. 2020). Managing tourism safety and experience in national parks is essential to reduce the effects of COVID-19 on people’s mental and physical health, and social cohesion.

Parks that have reopened and have experienced significantly increased numbers of visitors need to recognize how large numbers of visitors will affect tourism satisfaction and safety management. Thus, ensuring safe visit experiences during and after the pandemic remains an essential part of tourism experience management. Tourism may still be vulnerable to future health crises as well as the economic and social challenges that have resulted from the COVID-19 pandemic (Spenceley et al. 2021). Improving visitor experience and satisfaction in national parks and achieving tourism sustainability in both short-term and long-term development are of great importance.

Therefore, four research questions were proposed in our research:

1. What are the differences in visitor satisfaction level and the satisfaction determinants before and after COVID-19 in Banff National Park?
2. How have visitor demographic characteristics changed after the COVID-19 pandemic began?
3. What are the changes in visitor perceptions and frequency of visits towards the National Park after COVID-19 started?
4. What are visitor perceptions of the response to COVID-19 by the Town of Banff and how did they influence overall visitor satisfaction?

## Materials and methods

### Study area

**Fig. 1:**
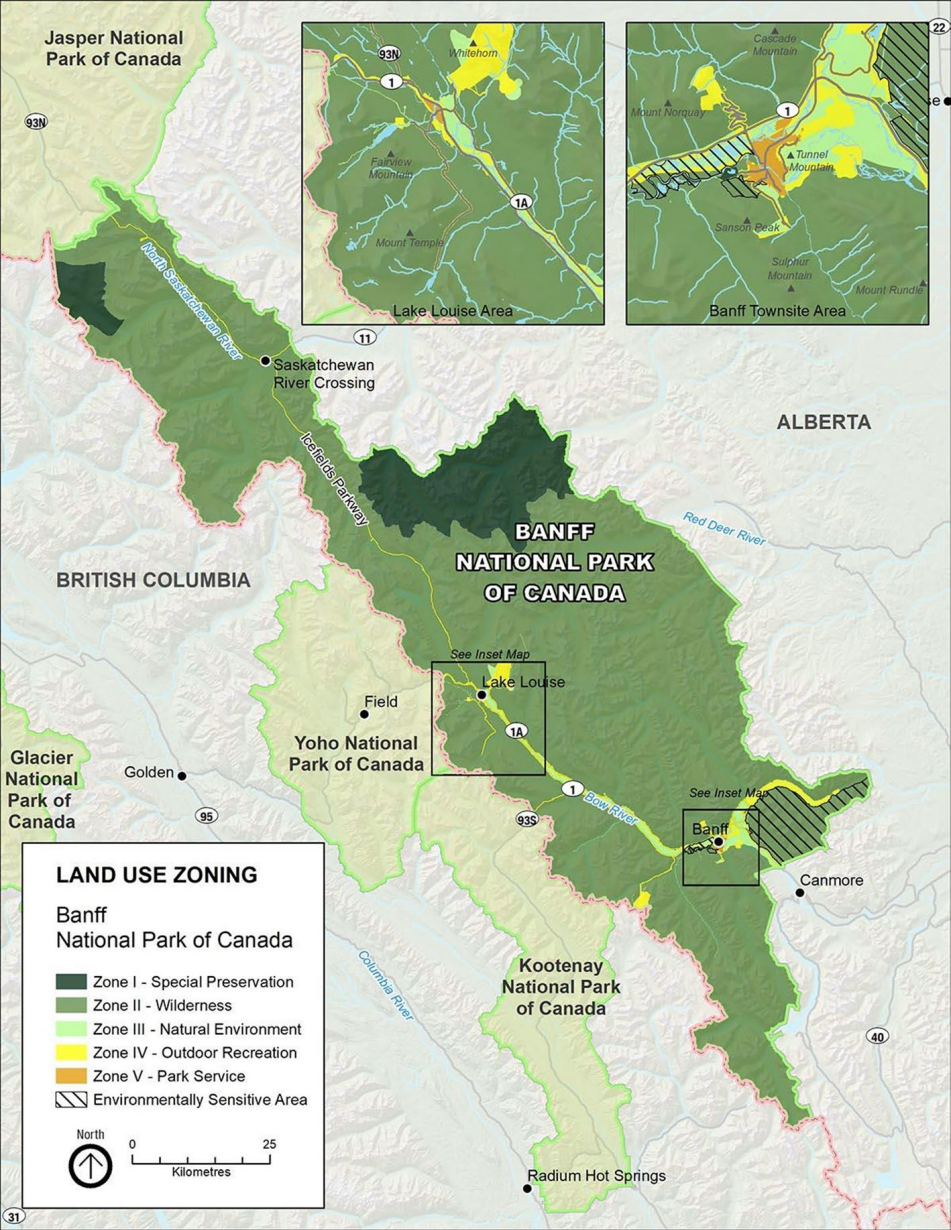
Land-use zoning of Banff National Park (Parks Canada 2021)

Banff National Park is located in the Canadian Rockies in southwestern Alberta, Canada. The Park encompasses 6,641 km^2^ of mountainous terrain and includes glaciers, coniferous forests, alpine landscapes, and scenic valleys (Banff and Lake Louise Tourism 2022; New World Encyclopedia 2022). Banff National Park has five management zones: Zone I (Special Preservation areas), Zone II (Wilderness areas), Zone III (Natural Environment areas), Zone IV (Outdoor Recreation areas) and Zone V (Park Services areas) (Fig. 1). This study was conducted in Zones III, IV and V.

Banff National Park was strategically selected as our study area for the following reasons. Firstly, as Canada’s first national park and a flagship for Parks Canada’s system of national parks, Banff National Park plays a significant role in providing unique natural characteristics and sophisticated visitor services for travelers all over the world. In 2019, Banff welcome more than 4 million visitors, which made it the most visited park in Canada (Parks Canada 2019). Secondly, the geographical location of Banff National Park makes it easily accessible from nearby cities. For example, it takes 1.5 h to drive from Calgary, Alberta to the Town of Banff, and there are shuttles/buses that run a direct route from Calgary to Banff. Banff is also close to other densely populated urban areas and cities such as Canmore (15-min drive) and Golden (1-h drive). Therefore, there was a significant number of local visitors from Canmore, Golden and Calgary who choose to visit Banff National Park during the pandemic. Lastly, Banff National Park was devastated by the COVID-19 pandemic in 2020 due to significantly fewer international visitors and reduced economic revenue. Therefore, Banff National Park was selected as our study area to further investigate the impacts of the COVID-19 on park tourism and management response.

### Questionnaire development

Questionnaires were developed with the purpose of collecting data about visitor demographics and satisfaction levels to examine whether these characteristics differed before and after COVID-19 started, and to investigate visitor perceptions about the response measures implemented by the Town of Banff. The questionnaire consisted of three sections and took each respondent approximately 15 min to complete.

In the first section, questions were asked about visitor demographic characteristics, in the second section about their satisfaction levels with their Park experiences, and their overall impression of the Park. The satisfaction items were selected based on a review of national park visitor experience research (Yuksel 2001; Tian-Cole et al. 2002; Rodger et al. 2015; Thapa and Lee 2017; Agyeman et al. 2019). Visitor satisfaction for all criteria and sub-criteria and visitor overall impression were measured using a five-point interval scale from very satisfied (1) to very unsatisfied (5). The third section of the questionnaire was designed to collect information on visitor attitudes about the nine COVID-19 pandemic restriction measures in place in Banff National Park (Table 5), and the change in visitor perceptions about the importance of national parks and other natural areas. Respondents were also asked about changes in the frequency of their visits to national parks before and after the COVID-19 pandemic.

Before the survey was carried out, multiple pre-tests were implemented with tourism staff and students. These pre-tests sought to identify and address problems such as ambiguous wording and to evaluate how long the questionnaire would take to administer. Pilot testing was also conducted on the first day of data collection to identify any site issues, and to finalize the approach for questionnaire distribution. The final questionnaire was reviewed and approved by the UBC Research Ethical Board. Data collection was conducted in July and December 2019, and October 2021. Paper-based questionnaires and instructions for accessing an online version of the questionnaire were distributed to visitors. In 2019, paper-based questionnaires were distributed in July and December; 291 and 450 completed questionnaires were returned, respectively. Due to concerns about COVID-19 virus transmission and visitor risk perceptions in 2021, potential respondents were provided in Banff National Park with the option of completing a paper-based questionnaire or an online version; 442 completed questionnaires were returned. There were no significant differences between paper-based and online responses, except for the age of respondents: a higher percentage of people (21 − 40 years-of-age) completed the online questionnaire than the paper-based one.

### Data processing and statistical analysis

A comparative analysis was conducted to investigate the impacts of COVID-19 on visitor satisfaction levels and demographics. Factor analysis with varimax rotation was performed to identify relevant visitor satisfaction dimensions. Pearson correlation analysis examined the relationships between visitor overall satisfaction and their satisfaction with the Park’s COVD-19 response measures. Stepwise regression tested two models (before and after COVID-19 started) and compared the influence of variables on overall visitor experience, and explored what improvements can be made to improve visitor overall satisfaction level during the pandemic.

To conduct a stepwise regression analysis, all of the nominal variables of visitor demographic characteristics were re-coded as dummy variables. The *gender* variable was recoded to female as 1 and male as 0, the *age* variable was recorded into three dummy variables, with age “20 and under” as a reference category. The *residential status* was recoded as local (from Alberta and British Columbia) and Canadian as a reference group, respectively. *Travel group composition* was recoded to travel alone as the reference group. For the *source of knowing Banff National Park* and *travel motivation*, knowing the Park from advertisement and pressure reduction were coded as the reference variables. For the *use of transportation*, private vehicle was coded as the dummy reference group. Lastly, the variable *duration of stay*, stay in Park for only one day was dummy coded as the reference group.

## Results

### Impacts of COVID-19 on Park visitor demographic characteristics

There were more female visitors after COVID-19 started and most visitors in both data collection periods were in the 21‒40 age category, and fewer visitors over 60 and younger than 20. The Park received fewer visitors from outside Canada in 2021 and more from local provinces and the rest of Canada (Table 1).

**Table 1:** Visitor demographic characteristics pre- and post- COVID-19 (*n* = 1183)

	Factors	Before COVID-19 started	After COVID-19 started
Gender	Male	54.4%	44.5%
	Female	45.6%	55.5%
Age	20 and under	17.3%	9.5%
	21‒40	50.6%	63.3%
	41‒60	21.8%	20.6%
	61 and over	10.3%	6.5%
Residency status	Local (Alberta and British Columbia)	41.4%	54.9%
	Canada (outside AB and BC)	12.7%	27.9%
	Outside Canada	45.9%	17.2%
Group composition	Alone	7.0%	10.6%
	Multi-person	93.0%	89.4%
Source (multiple choice)	Advertisement	6.9%	3.6%
	Webpage	9.9%	5.9%
	Brochure or magazine	3.6%	2.7%
	Friends or relatives	63.6%	78.8%
	Other	16.0%	8.9%
Reason for visit (multiple choice)	Pressure reduction	8.7%	11.6%
	Natural recreation	75.7%	79.0%
	Environment education	5.1%	4.3%
	Others	10.5%	5.1%
Number of times visiting	1	40.0%	33.3%
	2	11.4%	10.1%
	3	4.7%	6.5%
	Over 3	43.9%	50.1%

### Factor analysis on visitor satisfaction towards Park attributes (before and after COVID-19 started)

Overall, “Park attributes” generally received higher satisfaction ratings from visitors after COVID-19 started compared to data collected in 2019 (Table 6). “Landscape view” received the highest satisfaction rating both before and after COVID-19 started. After COVID-19 began, “Park basic infrastructure” such as trails, roads and pedestrian areas received a higher satisfaction level, followed by “Park services” and “natural characteristics”, and the “activities provided by the Park” received the lowest satisfaction level.

Factor analysis with varimax rotation was conducted to analyze visitor satisfaction for Park attributes (Table 2). When the satisfaction ratings for the 2019 and 2021 data were considered together, three dimensions were generated: Park infrastructure, Park services and activities, and Park natural characteristics, explaining 38.4%, 11.2% and 7.5% of the variance, respectively. The first factor incorporated roads, pedestrian walkways, and trails, and had the highest factor loading, indicating it is the most important component in the Park. Educational programs and interpretation systems provided by the Park had the highest loading for the second factor (Park services and activities). Lastly, flora and fauna had the highest factor loading in the third component, indicating their important role in determining this component load (Park natural characteristics).

**Table 2:** Comparison of factor analysis on visitor satisfaction level to Park attributes (*n* = 1183)

Overall PCA (KMO = 0.917)		Before COVID-19 Started (KMO = 0.872)		After COVID-19 Started (KMO = 0.932)	
National Park attributes and components	Factor loadings	National Park attributes and components	Factor loadings	National Park attributes and components	Factor loadings
Component I: *Park Infrastructure*		Component I: *Park Services and Activities*		Component I: *Park Services and Activities*	
Roads	0.789	Visitor centre	0.524	Visitor centre	0.499
Pedestrian sidewalks	0.763	Library	0.699	Garbage bins	0.446
Trails	0.770	Picnic and camping site	0.623	Kiosks	0.707
Hotels and hostels	0.613	Educational program	0.842	Library	0.771
Parking lots	0.713	Interpretation system	0.861	Souvenir stores	0.708
Washrooms	0.602	% of Variance: 31.3		Picnic and camping site	0.731
Signs	0.616	% of Cumulative: 31.3		Restaurants	0.558
Garbage bins	0.620	Component II: *Park Infrastructure*		Shopping mall	0.729
Restaurants	0.566	Road	0.840	Network and cell service	0.579
Network and cell services	0.425	Pedestrian sidewalks	0.811	Educational program	0.753
% of Variance: 38.4		Trails	0.733	Interpretation system	0.736
% of Cumulative: 38.4		Hotels and hostels	0.377	% of Variance: 44.2	
Component II: *Park Services and Activities*		Parking lots	0.509	% of Cumulative: 44.2	
Visitor center	0.570	% of Variance: 14.7		Component II: *Landscape and Infrastructure*	
Kiosks	0.562	% of Cumulative: 46.0		Landscape view	0.700
Library	0.778	Component III: *Convenient Facilities*		Roads	0.599
Souvenir store	0.557	Washrooms	0.699	Pedestrian sidewalks	0.569
Picnic and camping site	0.625	Signs	0.760	Trails	0.709
Shopping mall	0.547	Garbage bins	0.790	Hotels and hostels	0.441
Education programs	0.826	Kiosks	0.604	% of Variance: 8.2	
Interpretation systems	0.802	% of Variance: 7.6		% of Cumulative: 52.3	
% of Variance: 11.2		% of Cumulative: 53.7		Component III: *Park Facilities*	
% of Cumulative: 49.6		Component IV: *Recreational Facilities*		Parking lots	0.754
Component III: *Park Natural Characteristics*		Souvenir store	0.633	Washrooms	0.738
Flora	0.863	Restaurants	0.715	Signs	0.591
Fauna	0.869	shopping mall	0.730	% of Variance: 6.6	
Landscape view	0.613	Network and cell service	0.632	% of Cumulative: 58.9	
% of Variance: 7.8	% of Variance: 5.2	Component IV: *Park Natural Elements*			
% of Cumulative: 57.1	% of Cumulative: 58.9	Flora	0.857		
		Component V: *Natural Characteristics*	Fauna		0.879
		Flora	0.840	% of Variance: 4.8	
		Fauna	0.840	% of Cumulative: 63.7	
		Landscape	0.680		
		% of Variance: 4.8			
		% of Cumulative: 63.7			

Pre-COVID satisfaction ratings for Park attributes loaded onto five factors that accounted for 63.7% of the variance: Park services and activities, Park infrastructure, convenient facilities, recreational facilities, and natural characteristics. Among all of the Park attributes, the educational program, interpretation system, roads, pedestrian walkways, trails, signs, garbage bins, restaurants, shopping mall, flora, and fauna received high factor loadings over 0.7. Post-COVID satisfaction ratings of Park attributes loaded onto four components: Park services and activities, Park landscape view and infrastructure, Park facilities, and Park natural elements. The four components explained 63.7% of the total variance. Park attributes with high factor loadings were kiosks, library, souvenir stores, picnic and camping sites, shopping mall, educational program, interpretation system, landscape view, trails, parking lots, washrooms, flora, and fauna.

### Stepwise regression model comparison between pre- and post-COVID-19 in Banff National Park

The input independent variables included visitor satisfaction towards Park attributes and visitor demographic characteristics (Table 7). Natural characteristics and price level received the highest and lowest satisfaction level, respectively. This indicates that visitors tended to be more satisfied with Park natural characteristics such as flora and fauna and least satisfied with the price charged within the Park. In addition, there were more visitors that were very satisfied with the Park measures post-COVID-19 compared to pre-COVID satisfaction ratings.

Six independent variables were selected for the pre-COVID model (*R*^2^ = 0.472). Among them, satisfaction of Park facilities were first selected by the model and made a significant contribution to overall visitor satisfaction. Among all the visitor demographics dummy variables, means of transportation (plane or shuttle) was the only visitor demographic characteristic selected in the model. The effect size was calculated using Cohen’s *f* ^2^. The effect size of the regression model was equal to 0.89, which is considered to be large (Cohen 1992).

Five independent variables were selected for the post-COVID-19 stepwise regression model of overall visitor satisfaction (Table 3). Satisfaction of Park service was first selected by the model, representing 34.7% of the variance, followed by satisfaction towards Park facility, Park price reasonableness, and Park natural characteristics. Among all the input visitor demographic characteristics dummy variables, travel group composition as the only visitor demographic characteristic was selected by the model as the fifth independent variable. This regression model had a large effect size (*f*^2^ = 0.99).

**Table 3:** Variables in the stepwise regression model comparison analysis (*n* = 1183)

Period	Model	Variable entered	*R*	*R*^2^	Adjusted *R*^2^	Std. error of the estimate	Change statistics				
							*R*^2^ change	F change	*df*1	*df*2	Sig. F change
Before COVID-19 started	1	Satisfaction with Park facilities	0.524	0.274	0.272	0.470	0.274	120.486	1	319	< .001
	2	Satisfaction with Park price	0.618	0.382	0.378	0.434	0.108	55.298	1	318	< .001
	3	Satisfaction with Park activities	0.665	0.442	0.437	0.413	0.06	34.187	1	317	< .001
	4	Satisfaction with Park services	0.675	0.456	0.449	0.409	0.014	8.279	1	316	0.004
	5	Transportation—plane + shuttle	0.682	0.465	0.456	0.406	0.009	5.21	1	315	0.023
	6	Satisfaction with Park nature	0.687	0.472	0.462	0.404	0.007	4.295	1	314	0.039
After COVID-19 started	1	Satisfaction with Park services	0.589	0.347	0.345	0.489	0.347	170.228	1	321	< .001
	2	Satisfaction with Park facilities	0.642	0.413	0.409	0.464	0.066	35.945	1	320	< .001
	3	Satisfaction with Park price	0.676	0.457	0.452	0.447	0.045	26.254	1	319	< .001
	4	Satisfaction with Park nature	0.695	0.483	0.477	0.436	0.026	16.182	1	318	< .001
	5	Travel group—with others	0.706	0.498	0.490	0.431	0.014	9.148	1	317	.003

The stepwise regression pre-COVID-19 model explained 47.2% of overall satisfaction (Table 8). In this model, the standardized coefficient *β* values for all the satisfaction variables and means of transportation, (arrive by plane and shuttle), were positive, indicating that these variables were significantly and positively associated with overall Park satisfaction. More specifically, visitor who arrived the Park by plane and shuttle tended to have a higher overall satisfaction level compared to other means of transportation before COVID-19 started. Five independent variables were selected in the stepwise regression post-COVID model of visitor overall satisfaction level (Table 8). In this model, all the independent variables had a positive standard coefficient value, whereas the travel group composition was negative, indicating that those visitors who travelled alone to Banff National Park had higher overall satisfaction levels compared to those visitors travelled with others.

### Correlation analysis between overall visitor satisfaction and visitor experience to Park COVD-19 measures

Before correlation analysis was conducted, a descriptive analysis for visitor experience to Park COVID-19 measures was carried out (Table 9). All COVID-19 response measures had positive mean values from visitors. Of the nine response measures, # 9, “installed hand sanitizers and handed out free masks”, received the highest mean value, indicating that more visitors had a positive or very positive experience with this action. Overall, visitor positive experiences with the Park’s COVID-19 measures were reflected in the relatively high mean value of 4.12.

A correlation analysis was conducted to investigate the relationship between overall visitor satisfaction and visitor experiences with COVID-19 measures. The correlation analysis was based only on post-COVID data. All visitor experiences were positively correlated with visitor overall satisfaction (Table 4).

**Table 4:** Correlation between visitor experience to COVID-19 measures and overall visitor satisfaction (*n* = 442)

Visitor experience to COVID-19 measures	Visitor overall satisfaction level
Restricted indoor and outdoor recreation facilities and services to visitors	0.225**
Restricted playground use	0.153**
Restricted activities and major events with large gatherings cancelled	0.157**
Implemented mandatory mask bylaw	0.187**
Installed signs on trails and in the Park for COVID safety measures	0.142**
Provided helpline (phone/email) and delivery service	0.098
Provided online recreation program and activities	0.115
Created COVID complaint form for submitting concerns	0.088
Installed hand sanitizers and handed out free masks	0.128**
Overall, the Park’s COVID-19 response policies	0.219**

^*^ indicates *p* ≤ 0.05; ** indicates *p* ≤ 0.01, bold font indicates statistical significance

### Impacts of COVID-19 on visitor perception and behavior towards natural areas

**Fig. 2:**
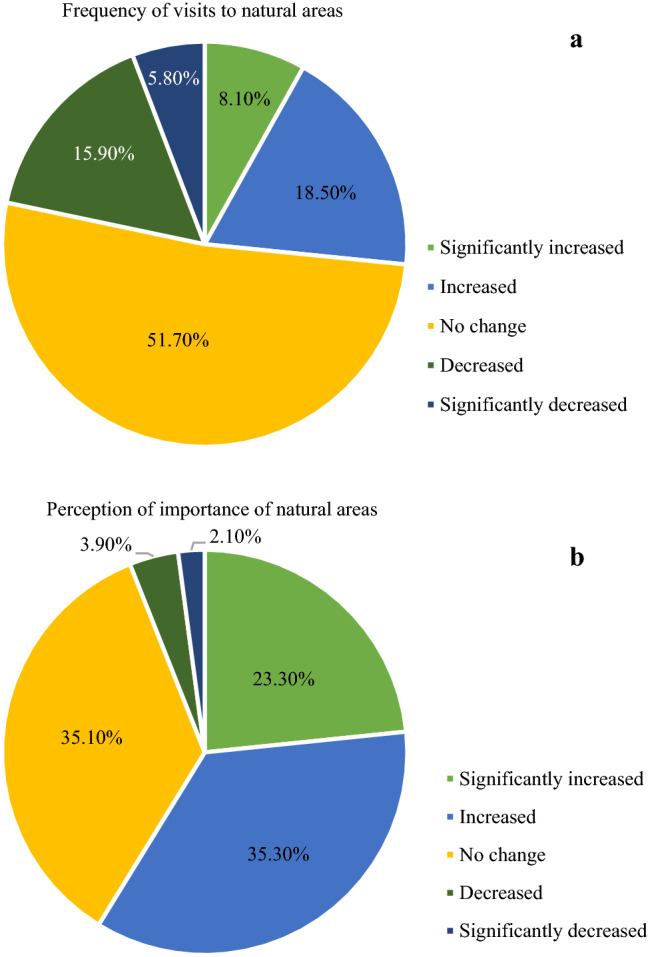
Impacts of COVID-19 on (a) visitor frequency of visits and (b) perception towards natural areas

This study also collected data on visitor perception and change in frequency of visits to national parks and other natural areas since COVID-19 started. More than half of the visitors increased or significantly increased their perception towards the importance of natural areas after COVID-19 started, and more visitors increased their frequency of visits (Fig. 2). Less than 10% of visitors decreased their perception of the importance of natural areas after the beginning of COVID-19.

## Discussion

### Changes in visitor demographic characteristics during the COVID-19 pandemic

Since COVID-19 started, the demographics of visitors to Banff National Park changed in different ways, the most important being visitor residency status. BNP had significantly fewer international visitors after the pandemic started, dropping by one-third, most likely due to international travel restrictions. With the decreasing number of international visitors, domestic visitation, especially from local/regional visitors from Alberta and British Columbia, increased significantly after the pandemic started. This is consistent with other COVID-19 research about national park visitation. Templeton et al. (2021) studied the influence of COVID-19 on US national park visitation and concluded that there were increasing numbers of regional and local visits. Moreover, the popularity of bus tours declined due to fewer international tourists, and more visits were made by single car trips by regional and local visitors (Templeton et al. 2021). Other studies observed that there were more domestic visitors during the pandemic, and that parks and other natural areas served as a setting where nature-based outdoor recreation could be engaged that balanced COVID-19 mitigation measures (e.g., physical distancing outside) with opportunities that supported mental and physical health (Geng et al. 2021; Rice and Pan 2021). This also explained the change of increasing pressure reduction as travel motivation and more visitors travelled alone after the pandemic started.

Another change in visitor demographic characteristics during the pandemic was visitor age composition. Although the 21 − 40 age category was the largest number for Banff visitation, there were fewer visitors ˂ 20 and > 60 after the pandemic started. This can be explained by the following. In 2020, over 56% of Americans between the age of 18 − 24 and 48.9% of Americans aged 25 − 49 reported symptoms of anxiety or depressive disorder after the COVID-19 pandemic started (Kaiser Family Foundation 2020). Natural areas, like BNP, provide opportunities to reduce and mitigate the negative mental impacts of the pandemic and related response policies. Secondly, people 61-year-old and over, a vulnerable age group, tended to follow stay-at-home recommendations and reduce the risk of being exposed to the virus. A consequence of this is that fewer older visitors have visited BNP since the pandemic started.

### Visitor satisfaction level and determinants comparison

*Visitor satisfaction level towards Park attributes before and after COVID-19.*

After COVID-19 started, Park attributes had higher satisfaction ratings. This finding is different than what has been found in research about COVID-19 impacts on consumer satisfaction levels. Although Mason et al. (2020) found that consumer satisfaction levels decreased from the beginning of the pandemic as a consequence of restrictions to limit the spread of the virus, our findings suggest that visitor satisfaction with Park response actions were positively correlated with overall satisfaction. Average visitor satisfaction with Park COVID-19 measures were positive, even those actions that restricted or limited activities. One explanation is that the decreased number of visitors addressed the pervasive issue of large numbers of visitors and overcrowding. In 2020, BNP received 4.1 million visitors. Visitors and residents reported issues such as being stuck in traffic, having trouble finding parking spots, battling long lines, overcrowding and overuse of the Park infrastructure (Ellis 2021). With international and domestic travel restrictions during the pandemic, BNP received fewer visitors and became less crowded compared to previous years (Government of Alberta 2022). Thus, visitors were able to easily access the Park services, facilities and infrastructure, and top tourist attractions with higher levels of satisfaction. Secondly, the Park received significantly more local and domestic visitors after the pandemic started, and over half of the visitors had made at least three previous trips to BNP before the survey. Local visitors usually have clear expectations about the quality of Park infrastructure, services, activities, and the natural characteristics of the Park prior to their visit (Simpson 2000; Lather et al. 2012). Research has shown that higher satisfaction ratings suggest that visitor expectations have been fulfilled: the tourism destination’s performance matches their expectations prior to the trip (Oliver 1993; Akama and Kieti 2003; Hui et al. 2007).

*Visitor satisfaction level factor analysis, determinants, and regression models comparison before and after COVID-19 started.*

Our stepwise regression models of overall visitor satisfaction determinants suggest that overall satisfaction before and after the COVID-19 pandemic was positively affected by the same Park elements but by different visitor demographic characteristics. Visitor satisfaction with Park facilities, services, natural characteristics and prices all had a significant impact which is consistent with previous research on visitor overall impressions and travel experiences (Lee et al. 2004; Rivera and Croes 2010; Dupeyras and MacCallum 2013; Rodger et al. 2015; Jovanovic and Ilic 2016; Thapa and Lee 2017; Virkar and Mallya 2018; Agyeman et al. 2019; Kim et al. 2019). Visitor demographic characteristics played a different role in determining overall visitor satisfaction before and after COVID-19 started. Visitors who travelled to BNP by plane or shuttle had significantly higher satisfaction ratings compared to those who came by other means of transportation. After the pandemic started, group composition was the only visitor demographic selected in the stepwise regression model due to its significant impact on overall visitor satisfaction. Visitors who travelled alone tended to be more satisfied compared to those who travelled with a group. This may be explained by the change of travel motivation after the COVID-19 started. Based on visitor demographics data, there were more people who visited BNP for pressure reduction potentially due to COVID-19 and associated restrictions, and fewer for natural recreation compared to prior to COVID-19. Before COVID-19 started, more visitors went to BNP for natural recreational activities in groups, whereas after, more people chose to visit BNP alone and use natural areas to reduce the mental and physical pressure caused by the pandemic and mitigate the negative effects caused by COVID-19 and self-quarantine with a more compelling travel motivation.

Moreover, our factor analysis results allow for the examination of the contribution of different Park elements to visitor satisfaction. Picnic and camping sites, parking lots, washrooms, and landscape views had increased weights in visitor satisfaction level in each factor group. Meanwhile, roads, pedestrian sidewalks, restaurants, and shopping malls had lower factor loadings, which means these Park attributes had lower weights in visitor satisfaction levels. During the COVID-19 pandemic and its associated restrictions, picnic and camping sites received higher variance of satisfaction compared to shopping malls and restaurants. Thus, prioritizing the management of Park picnic and camping sites, and other outdoor recreation and activities facilities is an approach that may increase visitor satisfaction in Banff National Park.

#### COVID-19 measures and associated visitor perceptions in Banff National Park

Visitor overall satisfaction was positively correlated with their experiences with Park COVID-19 restrictive measures. Although the Park’s response measures could have imposed additional constraints to visitors, all of the restricted measures received positive values from visitors. Seong and Hong (2021) found that visitor COVID-19 risk perceptions had a negative impact on their attitudes, subjective norms, and perceived visitor behavior, and suggested that COVID-19 risk perceptions acted as a constraint that negatively affected individual psychological characteristics when visiting national parks. BNP’s efforts to create a safe travel environment and provide a robust COVID-19 response system had positive impacts on visitor satisfaction, attitudes, and perceived behavior. The Park’s COVID-19 restrictions directly addressed risk factors and may serve to reduce visitor risk perceptions and increase their satisfaction with a safe travel experience. Visitors perceived risk and uncertainty from COVID-19 can influence their trip planning and activities, for example, a high COVID-19 risk perception may result in fewer trips and risk reduced behavior (Quintal et al. 2010; Seong and Hong 2021). This may indicate that travelling to a destination that has effective COVID-19 measures and low risk perception may allow them to increase their variety of trip activities, thus increase their travel experience. Effective and enhanced COVID-19 related public health restrictions and measures, such as those implemented in BNP, may increase visitor overall satisfaction.

Restrictions to indoor and outdoor recreation facilities and visitor services had the highest significant positive correlation with overall visitor satisfaction, followed by a mandatory mask bylaw. However, the online program and helpline provided, as well as the COVID-19 complaint form, was not significantly correlated with visitor satisfaction. One possible reason is that most visitors were not aware of the online services provided by the Park during the pandemic and hence these services were not utilized during their travel. Restrictions to facilities, and visitor services and mandatory mask bylaw directly affected visitors and lowered the risk of COVID-19 exposure.

Previous studies have shown that during the pandemic, and the resulting policies and measures, there had been increased demand for natural areas such as parks and green spaces to conduct safe outdoor activities and avoid the transmission of the disease (Grima et al. 2020; Geng et al. 2021; Mayen Huerta and Cafagna 2021; Rice and Pan 2021; Soga et al. 2021). This is consistent with our findings in terms of the frequency of visits and the perception towards natural areas. The most significant change after COVID-19 started was that over 58% of respondents reported increased importance of natural areas. This may be attributed to the benefits of parks on people’s mental and physical health under COVID-19 and associated restrictions.

### Challenges, opportunities, management implications and recommendations

It is critical for managers to effectively manage national parks to ensure positive and satisfying visitor experiences and safety. To achieve this, it is important to understand changes in park visitation, visitor demographics and satisfaction levels, and visitor perceptions of park COVID-19 response measures. This has been addressed by conducting a comparative analysis of visitation change before and after COVID-19 started and identifying management implications and recommendations to support sustainable Park management during future health crises.

COVID-19 has challenged-tourism management in national parks and has compelled Banff National Park management to find innovative ways to provide meaningful visitor experience while also protecting the safety and health of visitors, park workers and local communities from unnecessary risks (Town of Banff 2020). Maintaining the quality and variety of visitor services is critically important. More open public spaces and supportive infrastructure are also required for the Park to fully reopen under fewer international travel restrictions. Respondents were least satisfied with Park prices, especially local or domestic visitors, which raises the issue of how to balance visitor satisfaction with local prices and local business owners’ economic revenues. Banff National Park is inevitably one of the most popular tourism destinations, however, the cost to visit such as hotel charge, parking fee and restaurant price has raised concerns by visitors. In 2022, the average daily cost to Banff National Park in high season ranges from 1500 − 3100 CAD, which made Banff is one of the most expensive places to visit in Canada (Inspiring Travels Publishing Inc. 2022). The demographic characteristics of visitors are critical in determining the use and management of the Park (Takyi et al. 2018). With different compositions of international and national visitors, the traditional importance of activities such as guided interpretation and tour buses have decreased in popularity. According to Banff and Lake Louise Tourism (2022), local visitors conduct different activities during their travel to BNP and are more confident exploring the Park without guided support. Therefore, innovative activities were needed to serve the local and domestic visitors during the COVID-19 pandemic.

Lastly, it is not possible to predict how this pandemic will evolve, and what incidents will affect the development and recovery timeline (Spenceley et al. 2021). However, Park managers and other stakeholders can identify and examine the current situation and develop plans that can work. Determining the next steps for-tourism management in national parks is important; evidence shows that after the SARS crisis in 2003, a window of opportunity to improve tourism development opened, especially for nature-based tourism (Hong et al. 2020). New travel motivations to natural areas such as national parks and protected areas became evident with SARS (Sun et al. 2020). New tourism management strategies should be adopted to address increasing visitor numbers, such as maintaining standards for health and hygiene, and planning ways to distribute and disperse visitors spatially and temporally (Spenceley et al. 2021). Specific measures such as reservation requirements for top tourism sites and timed entry limitations may be required. Managing first-time visitors to national parks is also urgent and important: educational programs that can be delivered both online and in-person may help to increase their environmentally friendly awareness and behavior. One limitation of this study was that we lacked information on visitor perspectives on stay-at-home orders. Future research should be coordinated to examine the spatial pattern and distribution of visitors to Canadian Rocky Mountain parks. Future studies will also examine the impacts of distance from parks to nearby densely populated city downtowns and surrounding population size on national park visitation change.

## Conclusion

Management implications of answers to these questions were considered, and recommendations to support visitor safety and travel experiences under future potential health crises were provided. This research is a significant contribution to tourism management associated with national parks due to the following two aspects. First, there has been limited research comparing changes of visitor satisfaction levels and visitor demographics before and after COVID-19 started. Thus, our research provides management recommendations for both researchers and practitioners by combining the survey results from both before and after the pandemic. Lastly, the results and associated recommendations not only provide sustainable tourism management implications under the current pandemic but also to health crisis in the future.

Managing tourism in national parks under the COVID-19 pandemic was important to ensure visitor safety and health, increase visitor satisfaction, and achieve sustainable tourism management outcomes. Effective management requires understanding changing visitor demographics, travel satisfaction and associated determinants, and visitor perceptions of national parks before and after health crises start. This knowledge gap has been addressed by collecting and analyzing visitor data in Banff National Park on the impacts of COVID-19 on visitation and visitor satisfaction. Management implications and recommendations have been identified to aid in making changes to tourism services and facilities management, and to support effective tourism management approaches.

## Appendix

See Tables 5, 6, 7, 8, and 9

**Table 5:** COVID-19 pandemic restrictions measures in banff national park

Measure no	Restriction measures
1	Restricted indoor and outdoor recreation facilities and services to visitors
2	Restricted playground use
3	Restricted activities and cancelled major events with large gatherings
4	Implemented mandatory mask bylaw
5	Installed signs on trails and in the park for the COVID safety measures
6	Provided helpline (phone and email) and delivery service
7	Provided online recreation program and activities
8	Created COVID-19 complaint form for submitting concerns
9	Installed hand sanitizers and handed out free masks

**Table 6:** Comparison of the mean and standard deviation of visitor satisfaction level towards park attributes (*n* = 1183)

Before COVID-19 started			After COVID-19 started		
Park attributes	Mean	Std. Deviation	Park attributes	Mean	Std. Deviation
Landscape view	4.70	0.645	Landscape view	4.80	0.532
Trails	4.14	0.835	Trails	4.56	0.681
Signs	4.08	0.786	Road	4.36	0.741
Garbage bins	4.04	0.831	Pedestrian	4.32	0.827
Restaurants	4.02	0.886	Restaurants	4.30	0.853
Flora	3.96	0.896	Flora	4.30	0.813
Fauna	3.95	0.893	Garbage bins	4.28	0.820
Roads	3.88	0.976	Fauna	4.27	0.847
Pedestrian	3.87	0.908	Signs	4.19	0.875
Washrooms	3.87	0.905	Hostel	4.14	0.888
Shopping malls	3.77	0.879	Souvenir stores	4.00	0.902
Kiosks	3.72	0.83	Washroom	3.99	0.947
Visitor Center	3.67	0.834	Visitor center	3.95	0.881
Souvenir stores	3.67	0.841	Camping sites	3.94	0.908
Camping sites	3.64	0.834	Network and cell services	3.92	1.098
Network and cell services	3.61	1.007	Kiosks	3.90	0.888
Parking lots	3.58	0.948	Parking lots	3.89	1.010
Hostels	3.47	0.806	Interpretation system	3.88	0.891
Library	3.38	0.718	Shopping mall	3.77	0.941
Interpretation system	3.38	0.713	Library	3.65	0.899
Educational program	3.37	0.745	Educational program	3.63	0.884

**Table 7:** Visitor satisfaction with park attributes (*n* = 1183)

Satisfaction measures	Frequency percentage					Mean	Std. Deviation
	Very satisfied (%)	Satisfied (%)	Neutral (%)	Unsatisfied	Very unsatisfied		
*Before COVID-19*							
Natural characteristics	83.3	13.3	3.1	0.0	0.2%	4.80	0.502
Park facility	49.8	42.7	6.4	0.9%	0.2%	4.41	0.672
Park service	51.8	35.8	10.7	1.8%	0.0	4.38	0.745
Local community service	52.0	35.3	11.6	0.9%	0.2%	4.38	0.740
Activity and event	54.2	30.0	13.6	2.2%	0.0	4.36	0.798
Price reasonableness	16.0	27.8	35.8	17.3%	3.1%	3.36	1.042
*During COVID-19*							
Natural characteristics	92.0	6.8	0.7	0.2%	0.2%	4.90	0.379
Park facility	64.2	29.5	5.7	0.5%	0.2%	4.57	0.644
Park service	61.2	26.5	11.2	0.9%	0.2%	4.59	2.527
Local community service	56.0	25.7	17.4	0.2%	0.2%	4.45	1.619
Activity and event	50.1	28.9	18.9	1.6%	0.5%	4.27	0.852
Price reasonableness	26.2	30.1%	31.9%	9.3%	2.5%	3.68	1.040

**Table 8:** Stepwise regression models of visitor satisfaction determinants before pre- and after the post-COVID-19 started in Banff National Park (*n* = 1183)

	Model	Unstandardized coefficients		Standardized coefficients				95.0% Confidence Interval		Collinearity statistics	
			**B**	**Std. Err**	**Beta**	**t**	**Sig**	**Lower**	**Upper**	**Tolerance**	**VIF**
Model 1 before the COVID-19 started (R^2^ = 0.472)	1	(Constant)	2.648	.176		15.021	.000	2.301	2.994		
		**OFACILITY**	.432	.039	.524	10.977	.000	.354	.509	1.000	1.000
	2	(Constant)	2.447	.165		14.818	.000	2.122	2.772		
		OFACILITY	.338	.039	.409	8.767	.000	.262	.413	.892	1.121
		**OPRICE**	.183	.025	.347	7.436	.000	.135	.232	.892	1.121
	3	(Constant)	2.053	.171		12.004	.000	1.716	2.389		
		OFACILITY	.246	.040	.298	6.155	.000	.167	.324	.753	1.329
		OPRICE	.164	.024	.311	6.920	.000	.117	.210	.874	1.144
		**OACTIVITY**	.198	.034	.277	5.847	.000	.132	.265	.785	1.274
	4	(Constant)	1.943	.173		11.204	.000	1.601	2.284		
		OFACILITY	.174	.047	.211	3.735	.000	.082	.266	.539	1.855
		OPRICE	.152	.024	.288	6.379	.000	.105	.198	.847	1.181
		OACTIVITY	.188	.034	.262	5.556	.000	.121	.254	.775	1.291
		**OPSERVICE**	.116	.040	.159	2.877	.004	.037	.196	.566	1.767
	5	(Constant)	1.978	.173		11.438	.000	1.638	2.318		
		OFACILITY	.167	.046	.202	3.598	.000	.076	.258	.537	1.864
		OPRICE	.148	.024	.281	6.253	.000	.101	.195	.843	1.186
		OACTIVITY	.191	.034	.266	5.685	.000	.125	.257	.773	1.293
		OPSERVICE	.110	.040	.149	2.722	.007	.030	.189	.563	1.776
		**Transportation—Plane + shuttle**	.140	.061	.096	2.283	.023	.019	.260	.964	1.037
	6	(Constant)	1.471	.299		4.916	.000	.882	2.059		
		OFACILITY	.148	.047	.179	3.132	.002	.055	.240	.515	1.941
		OPRICE	.149	.024	.282	6.309	.000	.102	.195	.843	1.186
		OACTIVITY	.186	.033	.260	5.572	.000	.121	.252	.770	1.298
		OPSERVICE	.098	.040	.133	2.410	.017	.018	.177	.551	1.814
		Transportation—Plane + shuttle	.147	.061	.100	2.401	.017	.026	.267	.962	1.040
		**ONATURE**	.136	.066	.093	2.072	.039	.007	.266	.829	1.206
(R^2^ = 0.498) Model 2 after the COVID-19 started	1	(Constant)	2.464	.166		14.826	< .001	2.137	2.791		
		**OPSERVICE**	.477	.037	.589	13.047	< .001	.405	.549	1.000	1.000
	2	(Constant)	1.755	.197		8.897	< .001	1.367	2.143		
		OPSERVICE	.306	.045	.377	6.803	< .001	.217	.394	.597	1.676
		**OFACILITY**	.321	.054	.333	5.995	< .001	.216	.427	.597	1.676
	3	(Constant)	1.607	.192		8.367	< .001	1.229	1.985		
		OPSERVICE	.239	.045	.295	5.292	< .001	.150	.328	.547	1.828
		OFACILITY	.309	.052	.320	5.984	< .001	.207	.411	.595	1.680
		**OPRICE**	.136	.027	.230	5.124	< .001	.084	.188	.844	1.185
	4	(Constant)	.640	.305		2.099	.037	.040	1.240		
		OPSERVICE	.211	.045	.261	4.733	< .001	.124	.299	.534	1.872
		OFACILITY	.257	.052	.266	4.936	< .001	.155	.360	.559	1.790
		OPRICE	.139	.026	.236	5.374	< .001	.088	.190	.843	1.186
		**ONATURE**	.269	.067	.180	4.023	< .001	.138	.401	.812	1.232
	5	(Constant)	.807	.306		2.635	.009	.204	1.409		
		OPSERVICE	.206	.044	.254	4.658	< .001	.119	.293	.533	1.876
		OFACILITY	.261	.051	.270	5.077	< .001	.160	.362	.558	1.791
		OPRICE	.138	.026	.233	5.384	< .001	.088	.188	.843	1.186
		ONATURE	.239	.067	.160	3.573	< .001	.107	.370	.793	1.260
		Travel group—with others	− 940	.311	-.122	-3.025	.003	-1.552	-.329	.966	1.035

Dependent Variable: Overall Satisfaction. Bold font indicates statistical significance

OFACILITY: overall satisfaction towards park facility; OPRICE: overall satisfaction towards park price reasonableness; ONATURE: overall satisfaction towards park natural characteristics; OPSERVICE: overall satisfaction towards park services

**Table 9:** Frequency table for visitors’ experience towards park COVID-19 response measures (*n* = 442)

Response measures	Frequency percentage					Mean	Std. deviation
	Very positive	Positive	No impact	Negative	Very negative		
Measure 1	31.9%	27.3%	20.6%	17.3%	3.0%	3.68	1.177
Measure 2	22.6%	18.0%	49.9%	8.1%	1.4%	3.52	0.974
Measure 3	21.9%	22.2%	33.9%	18.5%	3.5%	3.41	1.123
Measure 4	38.3%	26.1%	21.9%	9.9%	3.7%	3.85	1.146
Measure 5	34.6%	30.7%	30.3%	2.8%	1.6%	3.94	0.951
Measure 6	27.3%	21.2%	48.5%	1.6%	1.4%	3.71	0.931
Measure 7	26.3%	21.0%	49.9%	1.8%	0.9%	3.70	0.912
Measure 8	23.3%	17.3%	56.1%	2.1%	1.2%	3.60	0.906
Measure 9	44.8%	34.2%	18.0%	1.8%	1.2%	4.20	0.877
Overall measures	39.5%	37.9%	18.5%	3.0%	1.2%	4.12	0.890

.
